# The evolution of alkaliphilic biofilm communities in response to extreme alkaline pH values

**DOI:** 10.1002/mbo3.1309

**Published:** 2022-08-16

**Authors:** Christopher J. Charles, Simon P. Rout, Brian R. Jackson, Sally A. Boxall, Sirwan Akbar, Paul N. Humphreys

**Affiliations:** ^1^ Department of Biological and Geographical Sciences, School of Applied Sciences University of Huddersfield Huddersfield UK; ^2^ Genesis Biosciences Unit P1 Capital Business Park, Capital Point Parkway Cardiff UK; ^3^ Bio‐imaging Facility, School of Molecular and Cellular Biology Faculty of Biological Sciences, University of Leeds Leeds UK; ^4^ Present address: Sulaimani Polytechnic University Sulaimani Iraq

**Keywords:** alkaline, alkaliphilic, biofilms, extremophiles, radioactive waste

## Abstract

Extremes of pH present a challenge to microbial life and our understanding of survival strategies for microbial consortia, particularly at high pH, remains limited. The utilization of extracellular polymeric substances within complex biofilms allows micro‐organisms to obtain a greater level of control over their immediate environment. This manipulation of the immediate environment may confer a survival advantage in adverse conditions to biofilms. Within the present study alkaliphilic biofilms were created at pH 11.0, 12.0, or 13.0 from an existing alkaliphilic community. In each pH system, the biofilm matrix provided pH buffering, with the internal pH being 1.0–1.5 pH units lower than the aqueous environment. Increasing pH resulted in a reduced removal of substrate and standing biomass associated with the biofilm. At the highest pH investigated (pH 13.0), the biofilms matrix contained a greater degree of eDNA and the microbial community was dominated by *Dietzia* sp. and *Anaerobranca* sp.

## Graphical abstract

Flocculants were used to seed biofilms in alkaline conditions (pH 11.0–13.0). Biofilms had pH and redox values lower than that of the external environment. Extracellular polymeric materials comprising the biofilm may play a role in these changes.

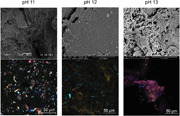

## 1 INTRODUCTION

Biofilm formation allows microorganisms to control their immediate environment by generating physical and chemical gradients at the micron scale. This control may lead to ecological advantages including protection against external stressors (Davey & O'Toole, 2000) such as antibiotics (Hall & Mah, 2017), disinfectants (Muazu et al., 2017), and pH (Charles, Rout, Laws, et al., 2017). Through global industrial activity and the prevalence of acid mine drainage, acidophiles and acidophilic biofilms have received considerable research focus (Aliaga Goltsman et al., 2015; Huang et al., 2016; Jiao et al., 2011). Alkaline biofilms have received less attention, with existing alkaline biofilm studies focused on either mildly alkaline systems (<pH 9.0) (Jones et al., 2015) or photosynthetic systems (Arp et al., 1999; Boomer et al., 2009).

A number of both natural (Grant, 2007) and anthropogenic (Gomes et al., 2016) alkaline environments have been studied as analogs for extra‐terrestrial environments (Blank et al., 2009) and radioactive waste disposal sites (Alexander et al., 2015). In anthropogenic alkaline sites where anoxic conditions are present, cellulose arising from plant matter is subject to chemical hydrolysis (Humphreys et al., 2010; Rout, Charles, Garratt, et al., 2015). This reaction results in the formation of cellulose degradation products (CDP) composed of a mixture of isosaccharinic acids (α and β stereoisomer) from cellulose and xyloisosaccharinic acid from the xylose (Almond et al., 2016). Despite the unique nature of these carbon sources planktonic cultures derived from anthropogenic alkaline soils (Kyeremeh et al., 2016) and neutral natural environments have both demonstrated the ability to metabolize these substrates up to, but not above pH of 11.0 (Rout, Charles, Doulgeris, et al., 2015; Rout, Charles, Garratt, et al., 2015).

Biofilms have been observed to form readily at pH ~12.0 on cellulosic materials emplaced within these anthropogenic alkaline sites (Charles et al., 2015). In this study, it was observed that the cellulose‐attached biofilm released a poly‐microbial, flocculant‐based community that was capable of survival at pH 12.0. Flocculants (flocs) are a type of biofilm that is typically a free‐floating composite of biofilm material and microbial cells. These flocs survived by using the extracellular polymeric substance (EPS) to attenuate the internal pH experienced by the resident microbial community (Charles, Rout, Laws, et al., 2017).

Within the present study, the ability of these poly microbial flocs to propagate biofilm formation on a sand matrix is explored. These biofilms were further exposed to influents at pH values of 11.0, 12.0, or 13.0, and microbial activity was determined through substrate metabolism and ATP content. 16S rRNA gene profiling was used to show the changes in community structure from a flocculant to surface biofilm existence under each pH value. The ability of these biofilms to modulate pH and eH was determined through micropH profiling and was visualized using a combination of scanning electron microscopy (SEM), with confocal laser scanning microscopy (CLSM) used to characterize biofilm components.

## 2 MATERIALS AND METHODS

### 2.1 Experimental biofilm systems

All experimental biofilms were grown on Ottawa sand (Sigma Aldrich Ltd) within a purpose‐built Biocell unit. Each Biocell unit held approximately 24 g of sand which was acid washed (1% v/v HCl) before use. The assembled Biocell sand units were sterilized by the passage of a sodium dichloroisocyanurate (1000 pm) solution for 2 h followed by rinsing with sterile nitrogen purged ultrapure water for 1 h. A microcosm was prepared, consisting of 50 ml of a previously described alkaliphilic floc forming community (Charles, Rout, Patel, et al., 2017) and 50 ml of CDP (Rout et al., 2014), brought to a working volume of 500 ml in anaerobic mineral media (B.S.I, 2005) and adjusted to pH11.0 using NaOH. The microcosm was made anaerobic through purging with nitrogen for 20 min The microcosm was continuously stirred (120 rpm) at 20°C and the contents were circulated through the Biocell at a rate of 0.35 ml min^−1^ using a peristaltic pump. This flow rate was chosen as it resulted in the passage of the entire volume (500 ml) of the subculture microcosm per day.

**Figure A1:**
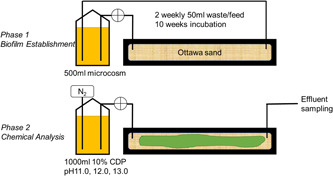
Overview of set up of biofilms and single pass experiment

After 2 weeks, 50 ml of fluid was removed from the microcosm and replaced with 50 ml of CDP and pH adjusted to 11.0 with 4M NaOH, this process was continued over 10 weeks at pH 11.0. For chemical analysis, Biocells were switched to a single pass configuration in which 1L of 10% v/v CDP diluted in anaerobic mineral media at either pH 11.0, 12.0, or 13.0 was passed through the biofilm at a rate of 0.05 ml min^−1^. These biofilms were run in duplicate and sampled every 2 days for 14 days. Anaerobic conditions were maintained through the connection of a nitrogen‐filled gas bag to the feedstock container. Abiotic controls were run alongside biotic experiments and operated under the same conditions using a sterile sand column. An overview of the experimental design is provided in Figure A1. A liquid sampling port was also employed to take liquid samples of the effluent. The pH of the effluent was determined using a calibrated probe and handheld unit (Mettler Toledo). The concentration of alpha (α), beta (β), and xylo (X) isosaccharinic acids were determined using high‐performance anion exchange chromatography with pulsed amperometry detection, where operating conditions are described in Rout et al. (2014).

### 2.2 Biofilm analysis

Following the duplicate flow‐through of the feedstock, the Biocell was disassembled under a nitrogen atmosphere to access the biofilm. The biofilm pH and redox profiles were taken through the sand‐attached biofilm using a 200 µm diameter micro pH electrode (Unisense) with internal reference and a 100 µm redox microelectrode with an external reference (Unisense) under a nitrogen atmosphere. Electrodes were connected to a single channel pH/redox meter supplied by the probe manufacturer (Unisense). The pH probe was calibrated using standard pH buffer solutions and tested against pH 11.0 and 12.0 solutions made up of NaOH. The redox probe was tested as per the manufacturer's instructions using pH buffer solutions saturated with quinhydrone. pH profiles and redox measurements were undertaken with a micromanipulator and stand supplied by the manufacturer (Unisense). Control profiles were taken through pH buffer or pH buffer amended with quinhydrone for the redox probe. Data were recorded using the SensorTrace suite (Unisense). Using a sterile spatula visible sections of biofilm were transferred to sterile Petri dishes for subsequent analyses. To estimate CFU within the biofilm, the removed biofilm material was washed once with phosphate‐buffered saline (pH 4.0) and then reconstituted in phosphate‐buffered saline (pH 7.0) to remove interference from excess alkalinity and salts. The samples were then processed using an ATP/biomass detection undertaken using a 3 M Clean‐Trace Biomass Detection Kit and Luminometer (3 M, UK). Following analysis ATP concentrations were quantified against a standard curve of known *Escherichia coli* K12 cell concentrations (10^2^−10^8^ CFU ml^−1^) to give cell number equivalents per gram of biofilm material utilized. Dry weight, volatile solids, and inorganic content of the biofilms were determined by methods outlined in BS ISO 6496:1999 (B.S.I., 1999). Crude EPS was extracted from biofilm using a multiple‐extraction method outlined by Ras et al. (2008) and is further explained in Charles, Rout, Patel, et al. (2017). Crude EPS extracts were measured for carbohydrate content via the phenol sulfuric acid method (Mecozzi, 2005), protein content via the Bradford assay (Bradford, 1976), lipid content via the methods of Bligh and Dyer (1959) with the DNA content measured via a Genova‐nano spectrophotometer at 260 nm (Jenway, Bibby Scientific). To purify the extracted EPS, dialysis was carried out using cellulose membrane dialysis tubing (14,000 typical molecular weight cut‐off, 33 mm width) against ultrapure water for 72 h with the water changed every 24 h. The protein and carbohydrate fractions were then isolated from the dialyzed EPS. Protein was precipitated via treatment with trichloroacetic acid (final concentration 14%) and the carbohydrate fraction precipitated via ethanol treatment as outlined in Marshall et al. (2001). Freeze‐dried fractions were broken down into constituent monomers using trifluoracetic acid (2 M) in a sealed pressure tube and heated for 2 h at 120°C. Following nitrogen purging and drying the monomers were reconstituted in ultrapure water and analyzed using HPAEC‐PAD, eluted at 10 mM NaOH for 20 min followed by a gradient of 83% 10 mM NaOH:17% 150 mM NaOH:1 M sodium acetate for 25 min and compared against a range of standards.

Biofilm material (~0.5 g) was removed from the sand and placed directly into a glass bead tube (Cambio). These tubes contained 450 µl phenol‐chloroform‐isoamyl alcohol (25:24:1) (pH 8.0) (Sigma), 450 µl CTAB extraction buffer, and 50 µl β‐mercaptoethanol (Fisher) (Griffiths et al., 2000) and then bead‐beaten for 10 min. After beating, samples were centrifuged for 1 min at 14,000 rpm and the aqueous phase was extracted and mixed with an equal volume of chloroform‐isoamyl alcohol (24:1) (Sigma). Samples were centrifuged again, the aqueous phase was extracted and the DNA/RNA precipitated with ethanol. The extracted nucleic acids were treated with DNase I to remove DNA, and cDNA was then generated using the Tetro cDNA Synthesis Kit (Bioline). Briefly: approximately 10–100 ng RNA was added to 1 μl random hexamer mix, 1 μl 10 mM dNTPs mix, 4 μl 5x RT buffer, 1 μl RiboSafe RNase inhibitor, 1 μl Tetro reverse transcriptase (200 U µl^−1^) and made up to a final volume of 200 μl using DNase/RNase free water. Samples were then placed in a thermocycler and incubated at 25°C for 10 min followed by heating to 45°C for 30 min. The reaction was terminated by incubating at 85°C for 5 min.

The V4 region of the 16S rRNA gene was then amplified using the universal eubacterial/archaeal primers 515F and 806R with the following overhangs 5′‐GTGCCAGCMGCCGCGGTAA‐3′ and 5′‐GGACTACHVGGGTWTCTAAT‐3′, respectively. PCR was completed using MyTaq red mix (Bioline) according to the manufacturer's instruction under the following conditions: initial denaturation (95°C, 1 min), followed by 35 cycles of denaturation (95°C, 15 s), annealing (60°C, 15 s), and extension (72°C, 10 s) followed by a final extension at 72°C for 5 min. Amplicons were then sequenced via a Miseq nano platform at 250 bp paired ends Microsynth with chimeric sequences identified and removed using the UCHIME algorithm (Edgar et al., 2011). OTU clustering based on 97% sequence similarity was carried out using Uclust within QIIME (v.1.9.1, Caporaso et al., 2010) and taxonomy assigned against the SILVA rRNA (database v.1111, Edgar et al., 2011; Quast et al., 2012). OTU clustering based on 97% sequence similarity was carried out using Uclust within QIIME (v.1.9.1) and taxonomy assigned against the SILVA rRNA (database v.1111).

### 2.3 Visualization of biofilm material

Two subsections of each biofilm exposed to influent at pH 11.0, 12.0, and 13.0 were removed from the Biocell and transferred aseptically to sterile Petri dishes for SEM and CLSM analyses. Subsections were initially fixed in 4% paraformaldehyde made up in PBS overnight at 4°C and then washed twice with PBS. Samples were then placed into a storage ethanol‐TRIS storage buffer described by Charles et al. (2015) and stored at −20°C before analysis. Fixed samples were dehydrated using a serial ethanol dilution of 25%, 50%, 75%, and 100% for 2 min per step. Samples were then dried and sputter coated via a gold‐palladium plasma (CA7625 Polaron, Quorum Technologies Ltd) and then viewed using a Quanta FEG 250 SEM and energy‐dispersive X‐ray spectroscopy (EDS) to investigate the morphology and elemental composition.

Structural components of the biofilms, including β‐1,4 and ‐1,3 polysaccharides, lipids and hydrophobic sites, β‐mannopyranosyl and β‐glucopyranosyl sugars, protein, total cells, and extracellular DNA were stained using the methodologies outlined in Charles, Rout, Patel, et al. (2017). These components were visualized using g a Zeiss LSM880 inverted confocal microscope, and image analyses were performed using Zen 2.1 (Zeiss Microscopy).

Live dead staining of flocs within the effluent was carried out using the LIVE/DEAD® *Bac*Light™ kit (Life Technologies) with flocs collected by centrifugation of 1 ml effluent on Day 14. After staining, flocs were washed twice with PBS and heat‐fixed onto a microscope slide for visualization and stored at −20°C before analysis. Fluorescence microscopy was carried out using an Olympus BX41 laboratory microscope (Olympus).

## 3 RESULTS

**Figure 1:**
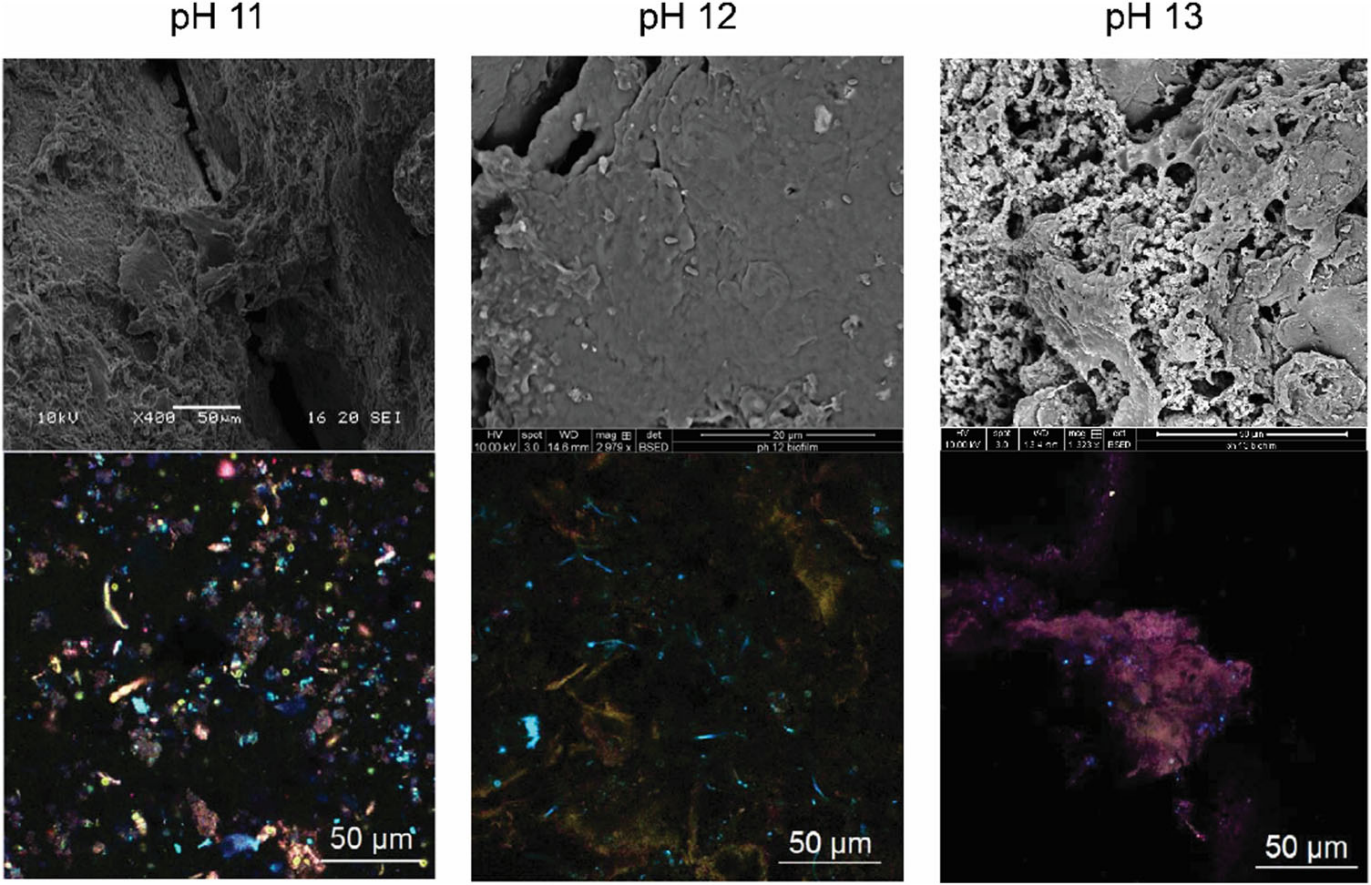
Scanning electron microscopy and confocal laser scanning microscopy images of biofilms formed on the surface of the sand within the Biocell following different influent pH values of substrate applied to the biofilm. Structural components of the biofilms include β‐1,4 and ‐1,3 polysaccharides (blue), lipids and hydrophobic sites (yellow), β‐mannopyranosyl and β‐glucopyranosyl sugars (red), protein (green), total cells and extracellular DNA (pink).

**Figure 2:**
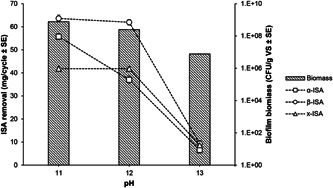
An increase in influent pH from 11.0 to 12.0 resulted in a reduction in the removal of the α‐ stereoisomer of isosaccharinic acid (ISA) and a slight reduction in biomass of biofilm. The increase in effluent pH to 13.0 resulted in a reduction in the removal of all forms of (ISA) and a further reduction in biofilm biomass.

**Figure A2:**
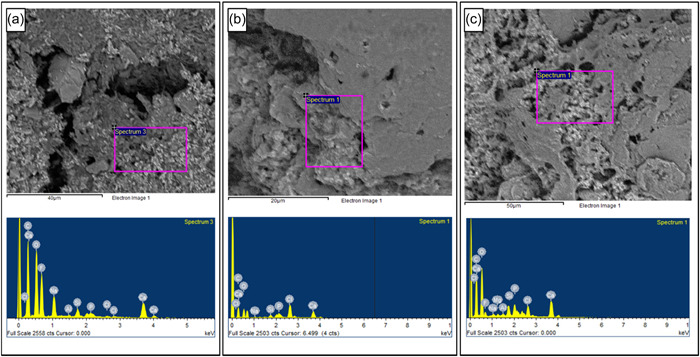
Scanning electron microscopy and EDX analysis of sand‐associated biofilms at (a) pH 11.0, (b) pH 12.0, and (c) pH 13.0 illustrating calcium carbonate inclusions

**Figure A3:**
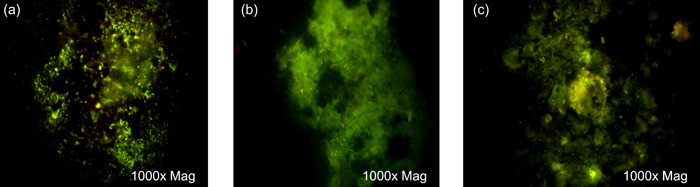
Microbial biomass in the biofilm effluent was contained within flocs of extracellular polymeric substances material (a, pH 11.0; b, pH 12.0; and c, pH 13.0). Images were generated through live dead fluorescent staining.

Biofilm survived on the surface of the sand at all influent pH values (Figure 1). The biofilms coated the surface of the sand grains with EDS analysis highlighting the presence of calcium, carbon, and oxygen, most likely calcium carbonate mineral inclusions punctuating the biofilm matrix (Figure A2). Isosaccharinic acids were consumed by the biofilm, however, as influent pH increased from pH 11.0 to pH 12.0, the removal of the α‐stereoisomer decreased, whereas the removal of the β isomer and xyloisosaccharinic acid remained stable (Figure 2). Following a further increase of pH from pH 12.0 to pH 13.0, a decrease in removal of all saccharinic acids was observed, which coincided with a 97% reduction in biofilm biomass when compared to that of the pH 12.0 biofilm with 7.9 × 10^6^ CFU/g volatile solids observed at pH 13.0. Despite this reduction in biomass, there was evidence of flocs sloughing from the biofilm and live/dead staining showed that live cells were present within these flocs (Figure A3). In addition to a reduction of biomass, changes were also observed in the community profiles as the influent pH increased.

**Figure 3:**
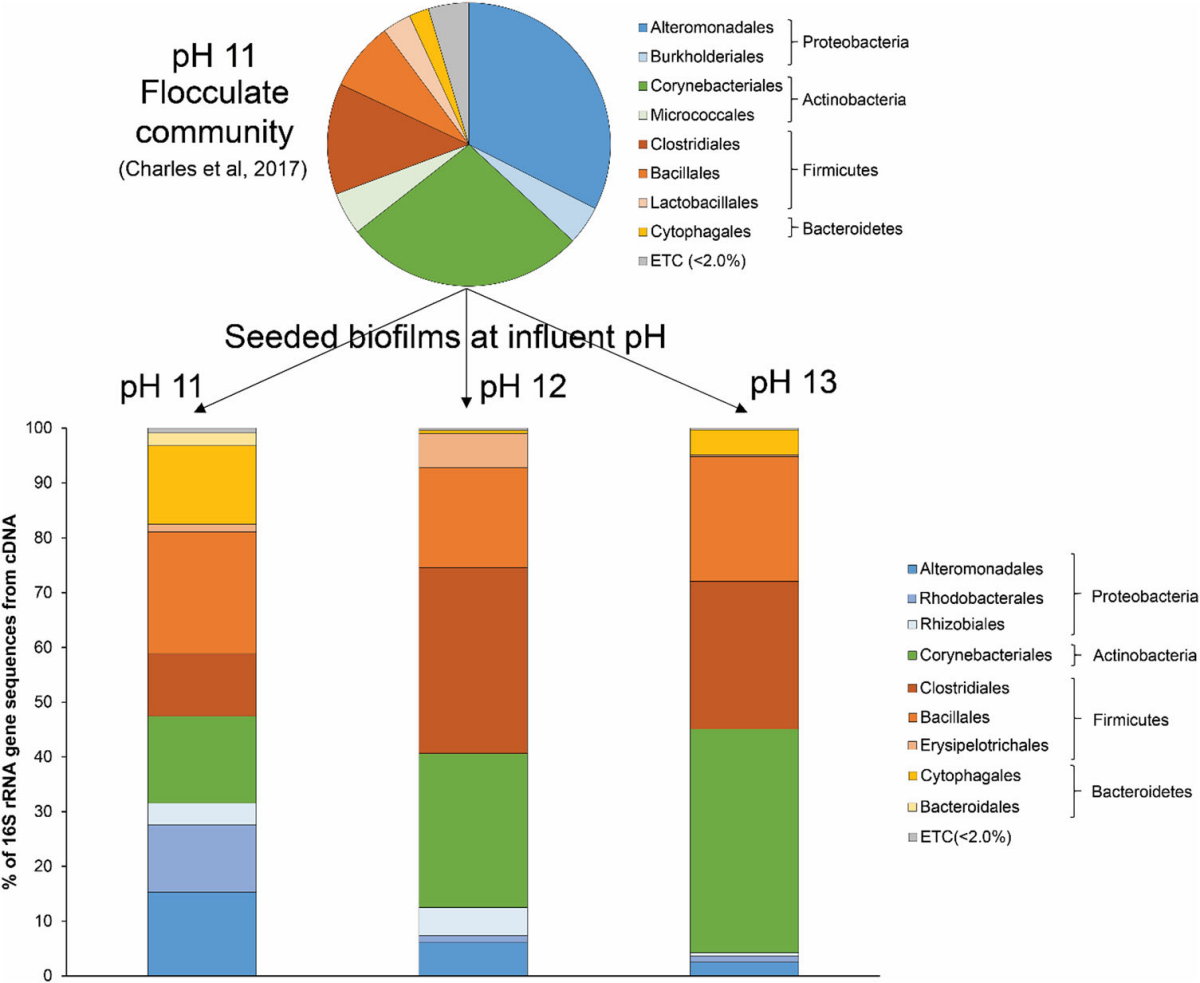
Taxonomic composition of 16S rRNA gene reads obtained from cDNA across the different influent pH values exposed, compared to the initial community of flocculant vector used to seed the biofilm. The increase in pH saw the community shift to prominence of Corynebacteriales, Clostridiales, and Bacillales at pH 13.0.

At pH 11.0, 16S rRNA gene community composition suggested that the biofilm was composed of OTUs from a range of taxonomic classes (Figure 3), where *Bacillales* (22.3%), *Corynebacterales* (15.9%), *Alteromonadales* (15.3%), *Cytophagales* (14.2%), *Rhodobacterales* (12.2%), and *Clostridiales* (11.5%), which were all active within the biofilm in similar proportions. The increase of pH from pH 11.0 to pH 12.0 resulted in an increased proportion of *Corynebacterales* (28.1%) and *Clostridiales* (33.9%), and taxa of the *Bacillales* fell slightly to comprise 18.2% of this community. Taxa of the *Alteramonadales* now comprised 6.2% of the OTUs observed, down from 15.3% at pH 11.0. A large drop in the detection of OTUs of the *Cytophagales* and *Rhodobacterales* was also observed, such that these now compromised <2% of the community at pH 12.0. A further increase in influent pH to pH 13.0 saw the proportion of *Corynebacterales* increase further to comprise 40.9% of the total community reads, exclusively of the genus *Dietzia*, those of *Clostridiales* (27.0%) and *Bacillales* (22.7%) remained consistent with those observed at pH 12.0. OTUs of the *Alteramonadales* fell again to comprise 2.5% of the community with the OTUs associated with *Cytophagales* increasing to 4.6% of the total reads.

**Figure 4:**
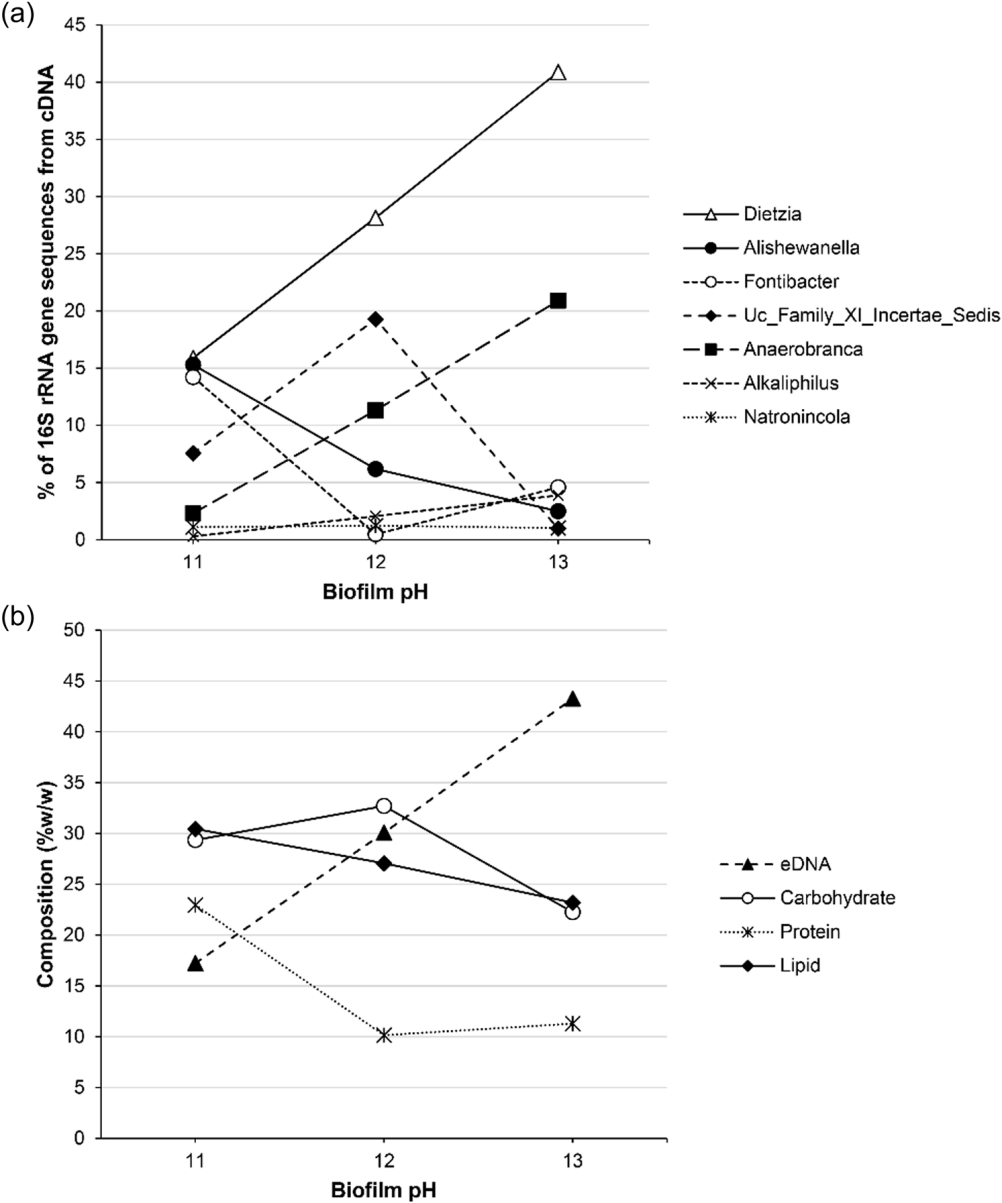
16S rRNA gene reads resolved to the genus classification from the biofilms exposed to pH 11.0, 12.0, and 13.0 effluents (a) which showed increases in *Dietzia* sp. and *Anerobranca* sp. These community changes coincided with an increase in eDNA and a decrease in protein, lipid, and carbohydrate within the extracellular polymeric substances material (b).

Within these OTUs further genus classification suggested that these groups were comprised of a single or limited number of genera. Within the *Corynebacterales*, *Cytophagales*, and *Alteramonadales*, only *Dietzia* sp., *Fontibacter*, and *Alishewanella* sp. were present respectively (Figure 4). The *Clostridiales* OTU was comprised of reads from 7 genera, three of which represented less than 1% of the total reads within each community. The greatest changes occurred with reads associated with *Anaerobranca* sp. and an uncultured clone of the Family XI Incertae Sedis. The *Anaerobranca* sp. represented 2.3% of the pH 11.0 biofilm community, this rose to 11.2% of the pH 12.0 community and 20.5% of the pH 13.0 community. Conversely, the uncultured clone represented 7.6% of the pH 11.0 community, which increased to 19.3% in the pH 12.0 community and fell to 1.0% of the pH 13.0 community. *Alkaliphilus* sp. also increased in proportion as pH increased, rising from 0.3% of the pH 11.0 community to 2.1% at pH 12.0, to 3.9% of the pH 13.0 community. The final genera of *Clostridiales* were represented by *Natronincola* sp., which remained consistent within each community, representing 1.0%–1.2% of the community.

**Figure A4:**
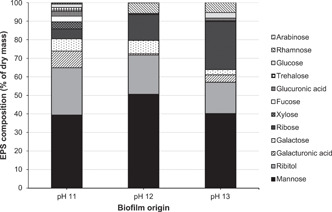
Relative composition of extracellular polymeric substances fractions obtained from biofilms operating at pH11, 12, and 13.

These changes in community size and structure also coincided with changes to the structural composition of the biofilm. At pH 11.0, extracellular DNA comprised 17.2% of the extracted biofilm material but represented 30.1% of the biofilm operating at pH 12.0% and 43.3% within the biofilm recovered from operation at pH 13.0. Conversely, the proteinaceous and lipid fractions of the recovered biofilm were both reduced following an operation at pH 12.0 and 13.0 compared to pH 11.0. Carbohydrates represented 29.4% of the EPS material recovered from the biofilm at pH 11.0, this increased to 32.7% when exposed to pH 12.0, but fell to 22.6% at pH 13.0. Despite this, the composition of the polysaccharide component of the EPS was largely consistent through the increasing pH, with core components being mannose and ribitol (Figure A4).

**Figure 5:**
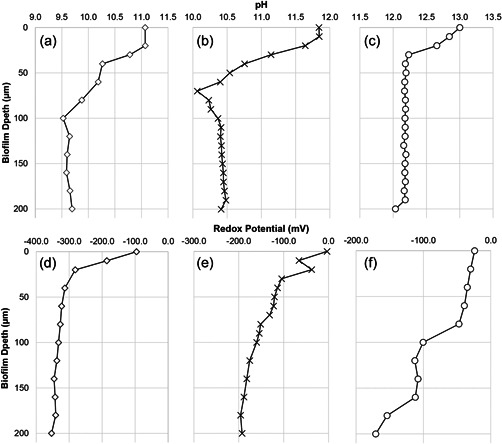
Microelectrode pH profiling of the pH 11.0 (a), 12.0 (b), and 13.0 (c) biofilms indicated a reduction in pH of 0.8−1.5 pH units from the liquid/biofilm interface to the surface/biofilm interface. This coincided with a reduction in redox potential through the biofilm at pH 11.0 (d), 12.0 (e), and 13.0 (f).

Microelectrode pH profiling through the EPS revealed sharp pH gradients over the initial 50−100 μm (Figure 5a–c). Biofilms at both pH 11.0 and pH 12.0 experienced a reduction in the internal pH by approximately 1.5 pH units across 100 μm, with internal biofilm pH remaining constant around pH 9.5 and pH 10.4 after that point respectively. Biofilm operating at pH 13.0 experienced a smaller drop of approximately 0.8 pH points across a 40 μm distance before reaching a stable internal pH of approximately pH 12.2. These “gradient” reductions in pH coincided with changes in the density of the biofilms at depth, where biofilms were denser at the sand surface compared to the exposed surface (data not shown). Redox profiles demonstrated that biofilm communities were able to generate a reducing environment at all pH values, however, at pH 13.0 the impact on internal redox potential was much reduced (Figure 5d–f).

## 4 DISCUSSION

The process of flocculation or aggregation is well studied with respect to biological wastewater treatment systems (Sheng et al., 2010). There has been recent recognition that aggregation is often a natural consequence of planktonic growth (Kragh et al., 2018) which can occur in response to stressors such as antibiotics (Corno et al., 2014) and be a precursor to biofilm formation (Melaugh et al., 2016). Within the present study, alkaliphilic biofilms were seeded on sand from polymicrobial flocs at high pH (11.0) and survived exposure to influents at pH 12.0 and 13.0. This demonstrates that flocs may be a key vector mechanism for the propagation of biofilms within hyperalkaline environments. The presence of flocs within the effluents of the single pass system also indicates that once a biofilm is formed on a surface, sloughing of floc material to seed further biofilms is likely to take place in extreme conditions.

From a wastewater treatment perspective, the switch between a floc and biofilm‐based existence is often associated with external stressors where EPS production plays a key role in this survival (Palmer et al., 2021). The biofilms generated within this study were resilient to their harsh exterior environment due to internal pH modulation linked to EPS production. As such, the formation of biofilm and propagation of biofilms is likely to be key to the survival of organisms within hyperalkaline environments. Irrespective of the influent pH the biofilms were exposed to, EPS was denser at the sand interface than that of the outward‐facing surface. This biofilm density appears to assist in the modulation of pH through the biofilm to the sand surface but may play a structural role in anchoring the biofilm to the sand surface (Desmond et al., 2022).

The EPS produced at each influent pH condition was composed of lipids, carbohydrates, proteins, and eDNA. Despite the similarities in composition across the pH conditions, there were also stark contrasts in the relative proportions of each component, which correlated with the changes observed within the community composition of each biofilm. Within a recent study by Aqueel and Liss (2022); it was suggested that the protein to polysaccharide ratio may play a role in influencing the sloughing of flocs at pH 7.0. Within the biofilms observed here, polysaccharides were consistently more abundant than proteins within the biofilms at all pH values. The monomer composition of the carbohydrate fraction of the EPS suggested that a core set of polysaccharides were present in each biofilm.

At pH 11.0, the biofilm community resembled that of the original flocculant community used to seed the biofilms on the surface of the sand., retaining several of the taxonomic groups observed. The biofilm formed at pH 13.0 saw a clear reduction in the amount and diversity of biomass with *Dietzia* being the most abundant genus and eDNA being the most abundant EPS component. Previous studies have noted their presence in alkaline and calcium‐rich environments (Duckworth et al., 1998; Kim et al., 2011; Yumoto et al., 2002). A recent review (Ibáñez de Aldecoa et al., 2017) of eDNA suggests that its role within a biofilm structure is likely to be numerous including the provision of structure, a source of nutrients, and DNA damage repair. With respect to structure, the chelation of metal ions may also be beneficial (Okshevsky & Meyer, 2015). Within this study, it is unclear whether *Dietzia* fulfills this role within the observed biofilms or whether the increased amounts of eDNA observed in the biofilm are a result of cell death and sorption to the sand at increased influent pH.

Species of the genus *Anaerobranca* also became a greater component of both the pH 12.0 and pH 13.0 biofilms compared to the initial flocs and the pH 11.0 biofilm. This genus is also noted for its extremophilic qualities (Prowe & Antranikian, 2001) and metabolism of several substrates through the production of alkali‐stable enzymes, which may assist in the metabolism of the ISA substrates provided within the influent (Thiemann et al., 2006). The retention of this genus and another unclassified OTU from the Family XI of the *Clostridiales* within the community coincided with the consistency of carbohydrates being detected in the EPS structure despite the loss of other genera following exposure to influent pH >11.0. The relative abundance of the carbohydrate component may indicate an ability to buffer the internal pH with polysaccharides possessing acid monomers (Mattos et al., 2001). The proteinaceous components of the biofilms formed were less within the pH 12.0 and pH 13.0 EPS compared with that of the biofilm formed at pH 11.0. Again, this coincided with the reduction in *Alishewanella* and *Fontibacter* sp. present within the community profiles. There is little literature evidence to support either of these genera being key to protein production, however, the ability of *Alishewanella* sp. to produce EPS material was recently observed in a previous study (Inaba et al., 2017).

Previous studies have indicated relatively short survival times for alkaliphilic flocculates above pH 12.0 (Charles, Rout, Patel, et al., 2017). Here we have demonstrated that within this short survival time if a surface is available for colonization, flocs act as a vector for biofilm formation, where these biofilms were stable at pH 13.0. As the pH increases the microbial community narrows to become dominated by members of the *Dietzia* and *Anaerobranca* genera which in turn coincides with increased dominance of eDNA and a reduction in proteinaceous EPS components. The findings here are specific to the microbial floc community used within the study, the demonstration of propagation and seeding of biofilms from flocs provide insight into a range of topics. The ability of microorganisms to colonize a concept geological disposal site for the UK nuclear waste inventory is one such example (N.D.A., 2010). The propagation of biofilms through flocs could be a primary route for intrusion into such a facility, where pH is likely to be 10.0–12.5 for a significant amount of time post closure (N.D.A., 2010). The formation of biofilms may influence radionuclide retention through influencing local geochemistry or pore blocking. From an astrobiology perspective, the presence of mafic minerals within the Martian environment could also present a potential source of alkaline conditions alongside the oceans of Europa (Kempe & Kazmierczak, 2002; McSween et al., 2006). Although there are other geochemical considerations with respect to these environments (Zolotov & Shock, 2003, 2004), the mechanisms of flocculation as a vector and EPS as a protectant from extreme conditions should not be overlooked.

## AUTHOR CONTRIBUTIONS

**Christopher J. Charles**: Conceptualization; data curation; formal analysis; investigation; writing—review and editing. **Simon P. Rout**: Conceptualization; data curation; formal analysis; writing—original draft. **Brian R. Jackson**: Investigation; methodology; visualization. **Sally A. Boxall**: Investigation; methodology; visualization. **Sirwan Akbar**: Investigation; methodology. **Paul N. Humphreys**: Conceptualization; formal analysis; funding acquisition; methodology; project administration; supervision; writing—original draft; writing—review and editing.

## CONFLICT OF INTEREST

None declared.

## ETHICS STATEMENT

None required.

## Data Availability

Raw data are presented within the figures. The 16S rRNA gene data are available in the NCBI sequence read archive under the BioProject accession number PRJNA339714: https://www.ncbi.nlm.nih.gov/bioproject/PRJNA339714, with the BioSample accession numbers SAMN05603705, SAMN05603706, and SAMN05603707 for the pH 11.0, pH 12.0 and pH 13.0 biofilms, respectively.
